# CTSL, a prognostic marker of breast cancer, that promotes proliferation, migration, and invasion in cells in triple-negative breast cancer

**DOI:** 10.3389/fonc.2023.1158087

**Published:** 2023-06-29

**Authors:** Lianmei Zhang, Yang Zhao, Jing Yang, Yaning Zhu, Ting Li, Xiaoyan Liu, Pengfei Zhang, Jingliang Cheng, Suan Sun, Chunli Wei, Junjiang Fu

**Affiliations:** ^1^ Department of Pathology, The Affiliated Huai’an No. 1 People’s Hospital of Nanjing Medical University, Huai’an, Jiangsu, China; ^2^ Key Laboratory of Epigenetics and Oncology, The Research Center for Preclinical Medicine, Southwest Medical University, Luzhou, Sichuan, China; ^3^ Department of Pathology, Taizhou People's Hospital of Nanjing University of Chinese Medicine, Jiangsu, China; ^4^ Department of Chemistry and Chemical Engineering, Hunan Institute of Science and Technology, Yueyang, Hunan, China; ^5^ NHC Key Laboratory of Cancer Proteomics, Department of Oncology, Xiangya Hospital, Central South University, Changsha, Hunan, China

**Keywords:** the CTSL gene, triple-negative breast cancer (TNBC), prognostics, cell proliferation, migration, invasion

## Abstract

**Introduction:**

In the world, the incidence of breast cancer has surpassed that of lung cancer, and it has become the first malignant tumor among women. Triple-negative breast cancer (TNBC) shows an extremely heterogeneous malignancy toward high recurrence, metastasis, and mortality, but there is a lack of effective targeted therapy. It is urgent to develop novel molecular targets in the occurrence and therapeutics for TNBC, and novel therapeutic strategies to block the recurrence and metastasis of TNBC.

**Methods:**

In this study, CTSL (cathepsin L) expression in tissues and adjacent tissues of TNBC patients was monitored by immunohistochemistry and western blots. The correlations between CTSL expressions and clinicopathological characteristics in the patient tissues for TNBC were analyzed. Cell proliferation, migration, and invasion assay were also performed when over-expressed or knocked-down CTSL.

**Results:**

We found that the level of CTSL in TNBC is significantly higher than that in the matched adjacent tissues, and associated with differentiated degree, TNM Stage, tumor size, and lymph node metastatic status in TNBC patients. The high level of CTSL was correlated with a short RFS (p<0.001), OS (p<0.001), DMFS (p<0.001), PPS (p= 0.0025) in breast cancer from online databases; while in breast cancer with lymph node-positive, high level of CTSL was correlated with a short DMFS (p<0.001) and RFS (p<0.001). Moreover, *in vitro* experiments showed that CTSL overexpression promotes the abilities for proliferation, migration, and invasion in MCF-7 and MDA-MB-231 cell lines, while knocking-down CTSL decreases its characteristics in MDA-MB-231 cell lines.

**Conclusion:**

CTSL might involve into the regulation of the proliferation, invasion, and metastasis of TNBC. Thus, CTSL would be a novel, potential therapeutic, and prognostic target of TNBC.

## 1 Introduction

Breast cancer (BC) is the most common malignancy in women worldwide. Relevant data show that there are about 2.3 million new cases of BC, and about 685,000 deaths worldwide in 2020 (1, 2). By 2040, the burden of breast cancer predicts to increase to more than 3 million new cases and more than 1 million deaths each year. While triple-negative BC (TNBC), a specific subtype of BC, accounts for 15% ~ 20% of all pathological types of breast cancer (3). This special subtype of BC is defined by the lack of the human receptors’ expression for estrogen (ER), and progesterone (PR) and the lack of amplification or expression for epidermal growth factor receptor 2 (HER2).

TNBC is the most difficult type of BC to treat at present, with the worst prognosis, the highest mortality, and the most prone to recurrence and metastasis. The treatment is almost only chemotherapy. Precision therapy is an important development direction for the future clinical treatment of TNBC patients. Although some progress has been made, there are still many difficulties. There is an urgent need to develop novel molecular targets in the occurrence and therapeutics for TNBC, and novel diagnostic and therapeutic strategies to block the recurrence and metastasis of TNBC.

Cathepsin L (CTSL, OMIM 116880), a member of the peptidase C1 family, is a lysosomal cysteine protease, which forms a dimer consisting of disulfide-linked heavy and light chains (4, 5). The cytogenetic location of the *CTSL* gene was mapped 9q21.33, which encodes a 333 amino acid length and 37564Da. In addition to playing important roles in intracellular catabolism, CTSL protein is involved in several pathological processes, such as myofibrillary necrosis (6, 7), and tumor progression/tumorigenesis (8–10), such as glioma cell migration and invasion by ionizing radiation-induced or X-ray-reduced manner (11, 12). CTSL was reported to proteolytically cleave the S1 subunit of spike protein on severe acute respiratory syndrome coronavirus 2 (SARS-CoV-2) or glycoprotein of Ebola virus (EboV) (13) for viral invasion into host cells (14–16). Gingival overgrowth and middle east respiratory syndrome (MERS) were also reported to associate with CTSL (17–19). CTSL is highly expressed in many malignant tumors, including gastrointestinal stromal tumors and metastatic bone tumors, identifying it as a new diagnostic or prognostic marker (20–22). Methyltransferase-like 3 (METTL3) enhanced the stability of CTSL mRNA m6A-IGF2BP2-dependent manners and promoted metastasis in cervical cancer cells (23). CTSL was reported to involve in the proliferation and invasion of breast cancer cells (24). In addition, studies in the breast cancer mouse model of PyMT have presented a strong influence of the CTSL/CTSB on lung metastasis and shown distinct effects on proteome composition in the metastatic lungs (25). However, it is unclear whether the regulation of CTSL expression is associated with metastasis, aggressiveness, and poor prognosis in TNBC patients.

In this research, we tested the CTSL role in TNBC and the correlations between CTSL expression and TNBC clinicopathology. The influence of CTSL on the biological function of the TNBC cell lines was analyzed by overexpressing and interfering with CTSL expression.

## 2 Materials and methods

### 2.1 Patient information and samples of TNBC

One hundred cases of paraffin-embedded breast cancer and paracancer tissues and thirty cases of fresh breast cancer and paracancer tissues were sampled from TNBC patients undergoing surgery in the Huai’an No. 1 People’s Hospital. The lymph node metastasis and received regional lymph node dissection were confirmed or suspected. All patients were diagnosed with TNBC by Immunohistochemistry (In the triple-negative breast cancer tissues, immunohistochemical staining of ER and PR in the nucleus and HER2 in the cell membrane were showed blue, which was interpreted as negative) and signed informed consent before surgery. The study protocol was approved by the Ethics Committee of Huai’an No.1 People’s Hospital.

### 2.2 Immunohistochemistry

We conducted immunohistochemistry (IHC) staining analysis to measure the protein expression of CTSL in TNBC tissues and adjacent normal breast tissues according to the standard immunoperoxidase staining procedure (26, 27). The Human TNBC tissues and adjacent normal breast tissues were embedded in paraffin and cut into 3mm thick slices, then placed on pre-coated slides. Placed them in an oven at 60°C for 1 h. Dewaxed and rehydrated: xylened for 3min, changed the solution again for 3min, then 50% xylene for 5min, 100% alcohol I-III for 1min each, 95% alcohol for 1 min, 75% alcohol for 1 min, ddH_2_O washed twice; Antigen retrieval was performed by heating the sample to 100°C in a 10 mM citric acid buffer; 1× PBS washed twice; ddH_2_O washed 3 times; 3%H_2_O_2_ incubated at room temperature for 10 min. Tissue sections were sealed with 5% BSA-1×PBST for 30min, incubated with CTSL antibody (1:100, Catalog No. ABIN1172740; Aachen, Germany), primary antibody at 4°C overnight, washed with 1×PBS twice, and incubated with secondary antibody at room temperature for 2h. The ABC (avidin-biotin-peroxidase complex) was incubated at room temperature for 2h, 1× PBS washed three times. The immunoreactivity was observed with DAB (diaminobenzidine), followed by reverse staining with hematoxylin. Dehydration and preservation: 75% ethanol for 30s; 100% ethanol for 30s; xylene for 30s; After sealed with neutral gum, covered with glass slides. The assessment was carried out by two independent pathologists in a double-blind method. Staining intensity and percentage of staining cells were scored respectively. The staining intensity score was 0 point: negative; 1 point: weak; 2 points: moderate; 3 points: strong. Staining cell percentage score 0: no cell staining; 1 point: staining cells <25%; 2 points: 25-50% cell staining; 3 points: 51-75% cells were stained; 4 points: >75% of the cells were stained. The final score for each tissue specimen is determined by multiplying the percentage of stained cells score by the staining strength score. The score of 0 was classified as negative, 1-4 as weak positive, 5-8 as positive, and 9-12 as strong positive. To distinguish the cut-off point of the CTSL expression level, receiver operating characteristic (ROC) curve analysis was used, and the score closest to the maximum Yoden index was used as the optimal cut-off value (27). Low CTSL expression was defined as samples with scores lower than cut-off values (score <6.3), while high CTSL expression was defined as samples with scores higher than cut-off values (score >6.3).

### 2.3 Western blotting

Firstly, the protein was extracted by 1×EBC lysate and 2xSDS, and the protein expression level was analyzed by Western Blot: 10 μl denatured protein samples were added to each well, electrophoresis with 10% SDS-PAGE, and protein separation completely was performed with 1 x running buffer at 100 V (28–30). The protein was transferred to the PVDF membrane at 100V for about 90 min. Then sealed the PVDF membrane at room temperature with 5% milk for 2h, and incubated the primary antibody with 2% milk at 4°C overnight. Primary antibodies included CTSL antibody (1:10000, Abcam Catalog No. ab200738), β-actin antibody (1:5000 Cell Signaling Technology), and HSP70 antibody (1:2000 Sigma USA). On the second day, PVDF (polyvinylidene fluoride) membrane was washed three times with 1×TBST, and the secondary antibody was incubated at room temperature for 1-2h. The secondary antibodies included anti-rabbit secondary antibodies (1:2000) and anti-mouse secondary antibodies (1:2000) which were purchased from Sigma (USA). The PVDF membrane was then cleaned with TBST. An imaging scanner was used to detect the strength of each cell membrane strip after TBST washing three times. Each experiment was repeated three times.

### 2.4 Online analysis for survivals

The Kaplan–Meier analysis for survivals was used to detect the prognosis of breast cancer patients including TNBC using the Kaplan-Meier Plotter (https://kmplot.com/analysis/index.php?p=service&start=1) (31, 32).

### 2.5 CTSL overexpression and knocking-down assays

MCF-7 cells and MDA-MB-231 cells were purchased from the American Type Culture Collection (ATCC), DMEM from Thermo Fisher Scientific, and Fetal Bovine Serum (FBS) from Pan Biotech, Germany. MDA-MB-231 and MCF-7 breast cancer cell lines were cultured in an incubator containing 5% carbon dioxide at 37°C. When the cell density reached 60%, the 900 ng CTSL overexpression plasmid was transfected with opti-MEM medium and Lipofectamine 3000, and the control group was transfected with a 900 ng empty vector. Then the transfected cells were cultured for another 24h.

The virus packaging process includes: designing CTSL target sequences (**Table 1**), constructing CTSL interference vector (pLV-hU6-CTSL shRNA2 (blue in **Table 1**, human):-hef1a-mNeongreen-P2A-Puro), 293T cells are often used for lentivirus packaging production in experiments because they can stably and efficiently package viruses. Then 293T cells were transfected, and the supernatant of the cells was collected, the viruses were harvested, the impurities were removed by centrifugation, the viruses were concentrated and purified, and the virus quality was tested. The MDA-MB-231 cells in good condition were seeded into a 24-well plate and the cells were planned at a density of 5x10^4^ cells/well. Then 500 μl DMEM medium is added to each well to ensure a fusion rate of 30-40% the next day. On the second day, the culture medium was replaced with DMEM containing 8µg/ml Polybrene, then the virus (MOI=20) was added to infect the MDA-MB-231 cells. The cell status was observed 12-24h after the virus infection. If the cells were in good condition, the DMEM medium was replaced within 24h. After 48h of virus infection, cells were observed under a fluorescence microscope to determine the efficiency of lentivirus infection.

**Table 1:** Human CTSL targeting sequences and its control sequence for knocking-down.

Name	Sequences (5’-3’)
NC shRNA	AAACGTGACACGTTCGGAGAACGAATTCTCCGAACGTGTCACGTTT
CTSL shRNA1	GAATTGCCTCAGCTACTCTAA
CTSL shRNA2	TGCCTCAGCTACTCTAACATT
CTSL shRNA3	AGGCGATGCACAACAGATTAT

### 2.6 Cell proliferation, migration, and invasion assays

6-well plates were applied to culture cell lines MDA-MB-231 and MCF-7 were cultured. When the cell density was about 60%, The cells were cleaned with PBS twice, digested with 0.25% trypsin, then added 1 ml serum-containing medium to stop digestion, centrifuged at 12000 rpm for 3 min, the supernatant was discarded, and 1ml serum-free medium was added to mix and counted with a bob-enumerating plate. On the CIM plate(monitoring cell growth), 13 μl serum and 17 μl serum-free medium, 100 μl cell suspension with a concentration of 2×10^4^ cells/ml were added to the upper chamber, and 165μl serum-containing medium was added to the lower chamber. On the CIM plate (monitoring cell migration), 30 μl serum-free medium and 100μl cell suspension with a concentration of 2×10^4^ cells/ml were added to the upper chamber, and 165 μl serum-containing medium was added to the lower chamber. CIM plate for monitoring cell invasion: First, apply glue (Matrigel matrix glue, purchased from BD Biosciences, Dilute with PBS at 1:40 ratio). After the upper chamber was gelled and the pores were dried for 1~2h, 30 μl serum-free medium and 100 μl cell suspension with a concentration of 2×10^4^ cells/ml were added to the upper chamber. 165 μl medium with 10% FBS was added to the lower chamber (28). The experimental group and the control group were provided with three secondary Wells. The cell proliferation, migration, and invasion were monitored by the real-time cell analyzer (xCELLigence RTCA DP, Roche, Germany) (28, 33).

### 2.7 Statistical analysis

The data were analyzed by SPSS 20 and GraphPad Prism 9. Pairwise comparisons of normally distributed data were analyzed by Student’s t-test or for multi-group comparisons, one-way analysis of variance (ANOVA). Counting data were tested by chi-square test. P < 0.05 (*), P < 0.01 (**), and P < 0.001 (***) are considered as statistical significances.

## 3 Results

### 3.1 CTSL expression in TNBC tissues is higher than that in the matched paracancerous tissues

**Figure 1:**
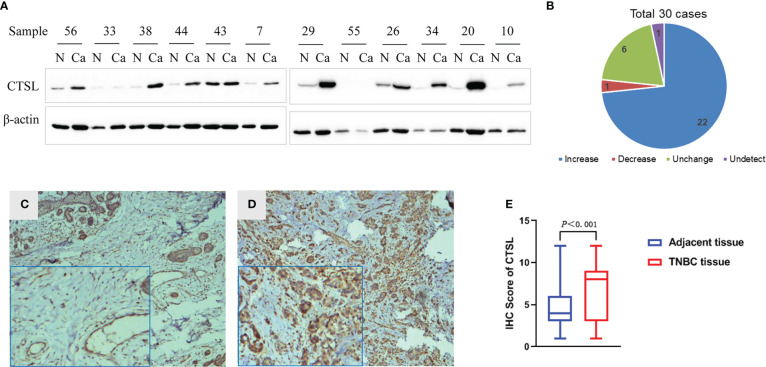
CTSL expression in TNBC by western blotting (WB) and immunohistochemistry (IHC). **(A)** Representative figures of CTSL expression in 30 cases of TNBC and the matched adjacent tissues by western blotting (WB), the expression of CTSL in TNBC tissues is mostly higher than that in the matched adjacent tissues. **(B)** Statistical analysis of CTSL expression in TNBC and the matched adjacent tissues of WB). **(C)** The CTSL expression is low in paracancer tissues than that of TNBC tissues (IHC 10×). **(D)** CTSL is more highly expressed in TNBC tissues than that of paracancer tissues (IHC 10×). Enlarged images are shown in the left corners of panels C and D, respectively. **(E)** Statistical analysis of CTSL expression in TNBC and the adjacent tissues (IHC).

To explore the expression of CTSL in TNBC, one hundred of Paraffin-embedded TNBC and paracancer tissues were collected for immunohistochemical, and thirty fresh TNBC tissues and the matched paracancer tissues were for western blotting analysis. The results showed high expression of CTSL in 22 cases of TNBC tissue, low expression in one case of TNBC tissue, and no significant difference in the expression of CTSL in six cases of TNBC tissues when compared with adjacent tissues. In one case, CTSL protein expression was not detected (**Figures 1A, B**). Therefore, the protein expression levels of CTSL were significantly higher in TNBC tissues than that in the matched adjacent tissues (**Figure 1B**, P<0.001). Meanwhile, the immunohistochemical results also showed that CTSL is more highly expressed in TNBC than that in the matched adjacent tissues (**Figures 1C–E**).

### 3.2 Analysis of the correlations between CTSL expression and clinicopathological characteristics in patients for TNBC

The information of one hundred patients with TNBC cancer was collected for analyzing the correlations between CTSL expression and clinicopathological data. In 100 cases of TNBC, 85% was positive for CTSL. We thus divided them into CTSL high expression group (n=59) and CTSL low/no expression group (n=41) based on the CTSL immunohistochemical score. Comparing the clinicopathological data, the results showed that the CTSL expression was associated with differentiated degree, tumor size, TNM stage, and lymph node metastasis in TNBC patients (**Table 2**, blue colors). Thus, these data demonstrated that CTSL might play clinicopathological characteristics roles including metastasis in TNBC patients.

**Table 2:** CTSL expression and clinicopathological characteristics in TNBC patients (n=100).

	High expression group (n=59)	Low/no expression group (n=41)	P value
Age(year)			
>50	29	27	0.097
≤50	30	14	
Differentiated degree			
high	19	22	0.032
Moderate-low	40	19	
TNM Stage			
I-II	30	31	0.013
III	29	10	
Size(cm)			
> 2cm	47	25	0.041
≤ 2cm	12	16	
Lymphatic metastasis			
Yes	39	15	0.004
No	20	26	

### 3.3 CTSL expression is a poor prognostic marker for BC

**Figure 2:**
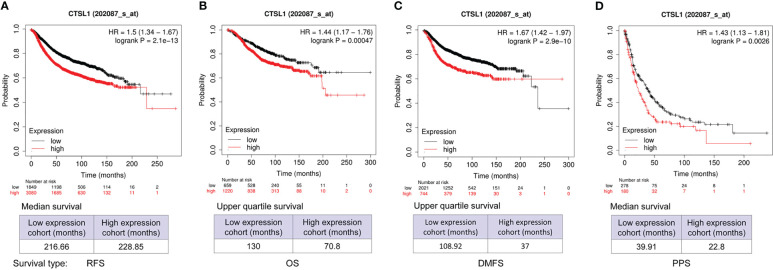
CTSL expression affects the survivals of BC patients for recurrence-free survival (RFS), overall survival (OS), and post-progression survival (PPS). **(A)** The clinical correlations between CTSL level and RFS (p<0.001). **(B)** The clinical correlations between CTSL level and OS (p<0.001). **(C)** The clinical correlations between CTSL level and DMS (p<0.001). **(D)** The correlations between CTSL level and PPS (p<0.001). The Kaplan–Meier analysis for survivals was used to detect the prognosis of breast cancer patients using the Kaplan-Meier Plotter (https://kmplot.com/analysis/index.php?p=service&start=1).

We analyzed the correlations between CTSL expression and outcomes of survivals including recurrence-free (RFS), overall (OS), distant metastasis-free (DMFS), and post-progression (PPS) in BC by online databases. The results showed that a high level of CTSL is significantly correlated with a short RFS (**Figure 2A**, p<0.001), OS (**Figure 2B**, p<0.001), DMFS (**Figure 2C**, p<0.001), PPS (**Figure 2D**, p=0.0025) in breast cancer. Hence, CTSL expression could be a poor prognostic marker for survival in BC.

### 3.4 Prognosis results of CTSL expression in TNBC

**Figure 3:**
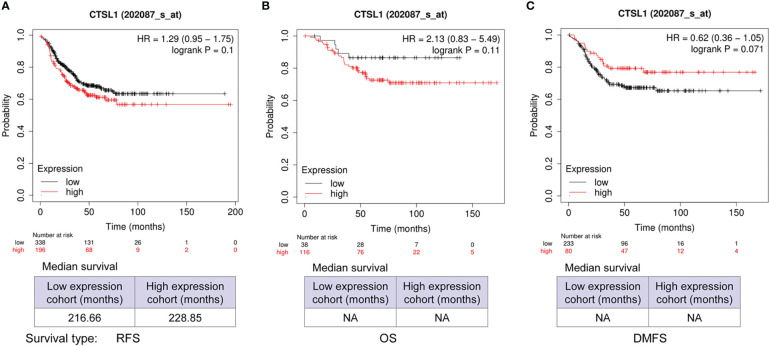
Prognosis analysis of CTSL expression in TNBC for recurrence-free survival (RFS), overall survival (OS), and post-progression survival (PPS). **(A)** The correlations between CTSL expression and RFS (p=0.1). **(B)** The correlations between CTSL expression and OS (p=0.11). **(C)** The clinical correlations between CTSL level and DMFS (p=0.071). The Kaplan–Meier analysis for survivals was used to detect the prognosis of TNBC patients using the Kaplan-Meier Plotter (https://kmplot.com/analysis/index.php?p=service&start=1).

We further analyzed the correlations between CTSL expression and outcomes of survivals including RFS, OS, and DMFS in TNBC by Kaplan–Meier analysis. The results revealed that a high level of CTSL is correlated with a short RFS (**Figure 3A**, p=0.1), and OS (**Figure 3B**, p=0.11) in TNBC. While the results revealed that a high level of CTSL is correlated with a long DMFS (**Figure 3C**, p=0.071). Hence, CTSL expression might be a poor prognostic marker for RFS and OS in TNBC.

### 3.5 Prognosis results of CTSL expression in BC with lymph node-positive

**Figure 4:**
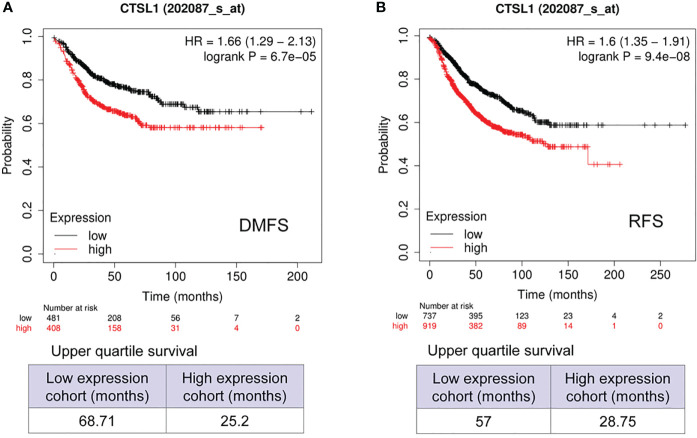
Prognosis analysis of CTSL expression in BC with Lymph node positive for distant metastasis-free survival (DMFS) and recurrence-free survival (RFS). **(A)** The correlations between CTSL level and DMFS (p<0.001). **(B)** The correlations between CTSL level and RFS (p<0.001). The Kaplan–Meier analysis for survivals was used to detect the prognosis of breast cancer patients using the Kaplan-Meier Plotter (https://kmplot.com/analysis/index.php?p=service&start=1).

We further analyzed the correlations between CTSL expression and outcomes of survivals of DMFS and RFS in breast cancer with Lymph node-positive. The results revealed that a high expression of CTSL is correlated with a short DMFS (**Figure 4A**, p<0.001) and RFS (**Figure 4B**, p<0.001). Hence, CTSL expression could be a poor prognostic marker in breast cancer with lymph node-positive.

### 3.6 Overexpression of CTSL promotes cell proliferation, migration, and invasion of MDA-MB-231 and MCF-7

**Figure 5:**
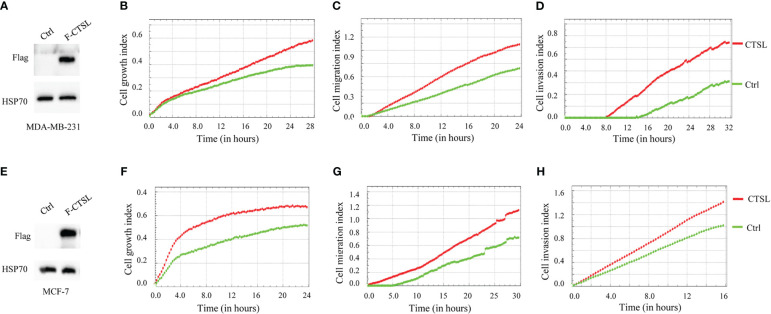
CTSL expression affects proliferation, migration, and invasion. **(A)** CTSL is successfully overexpressed in the indicated MDA-MB-231 cells. **(B)** Overexpression of CTSL increases the proliferation in the indicated MDA-MB-231 cells. **(C)** Overexpression of CTSL enhances the migration ability in MDA-MB-231 cells. **(D)** Overexpression of CTSL increases the invasion ability of MDA-MB-231 cells. **(E)** CTSL is successfully overexpressed in the indicated MCF-7 cells. **(F)** Overexpression of CTSL increased the proliferation in MCF-7 cells. **(G)** Overexpression of CTSL enhances the migration ability in MCF-7 cells. **(H)** Overexpression of CTSL enhances the invasion ability of MCF-7 cells. Flag antibody was used for the detection of CTSL overexpression. Breast cancer cell lines MDA-MB-231 and MCF-7 were used for CTSL overexpression and then to monitor the proliferation, migration, and invasion by the real-time cell analyzer (xCELLigence RTCA DP, Roche, Germany).

To further analyze the influence of CTSL expression on the behaviology of TNBC cells, we constructed a Flag-tagged CTSL overexpression vector, and transfected CTSL overexpression plasmid into MDA-MB-231 and MCF-7 respectively, which showed successful overexpression by western blotting with Flag antibody (**Figures 5A, E**). Then, we analyzed their proliferation, migration, and invasion capacities and the results revealed that overexpression of CTSL significantly enhanced the capacity of proliferation, migration, and invasion in MDA-MB-231 cell lines (**Figures 5B–D**) and MCF-7 cell lines (**Figures 5F–H**), respectively.

### 3.7 Downregulation of CTSL inhibits proliferation, migration, and invasion of MDA-MB-231 cell lines

**Figure 6:**
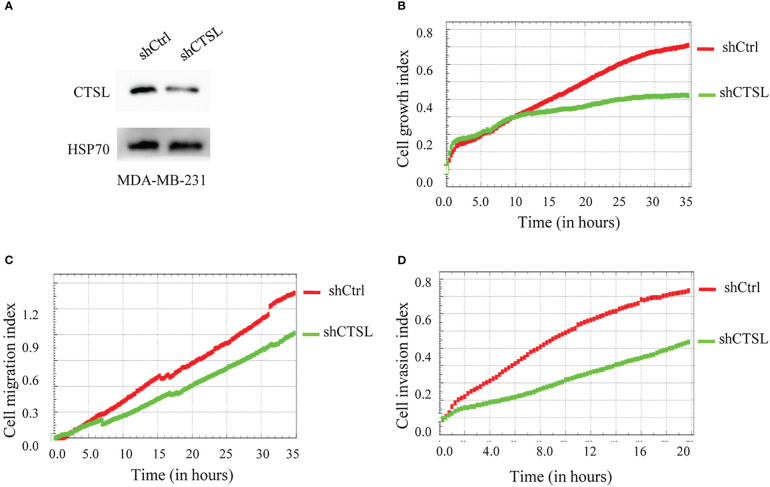
CTSL knocking-down affects the proliferation, migration, and invasion of MDA-MB-231 cells. **(A)** CTSL is successfully knockdown in MDA-MB-231 cells by western blots (CTSL shRNA2). **(B)**: knockdown CTSL decreases the proliferation in MDA-MB-231 cells. **(C)** knockdown CTSL decreases the migration ability of MDA-MB-231 cells. **(D)** knockdown CTSL decreases the invasion ability in MDA-MB-231 cells. Breast cancer cell line MDA-MB-231 was used for CTSL knocking-down and then to monitor the proliferation, migration, and invasion by the real-time cell analyzer (xCELLigence RTCA DP, Roche, Germany).

On the other hand, we constructed three CTSL knocking-down vectors (**Table 1**, in blue color), and one of them (CTSL shRNA2) was knocked down well in TNBC cell lines MDA-MB-231 (**Figure 6A**). We then analyzed their proliferation and migration and invasion capacity. The results revealed that CTSL knocking-down could significantly inhibit the capacity of proliferation and migration and invasion in TNBC cancer cells MDA-MB-231 (**Figures 6B–D**).

## 4 Discussion

Proteases were reported to involve several stages of tumorigenesis and progression and act as prognostic markers (34–36). CTSL, a member of the papain superfamily of cysteine protease, is involved in the proliferation, invasion, and metastasis of various cancers. For example, relevant studies have found the expression of CTSL level in glioblastoma multiforme (GBM) tissues is higher than that in healthy brain tissues (37), which is a proteolytic enzyme as a potential therapeutic target in this tumor disease. CTSL was reported to involve the growth and invasion of human ovarian cancer cells (38). Down-regulation of CTSL could significantly inhibit the growth and invasion of ovarian cancer cells and sensitized these cells to chemotherapy (38, 39). CTSL enzyme activity or expression was significantly higher in many cancers, including highest in kidney and testicular tumors, then higher in the lung, adrenal, bladder, breast, colon, ovary, prostate, and thyroid cancers than in normal controls (21, 40). This suggests that the upregulation of CTSL expression may be closely related to tumor progression.

Thus, in addition to CTSL acting as one of the SARS-CoV-2 receptors, there is increasing evidence that proteolytic enzymes play important roles in cancers. Several CTSL inhibitors have been developed and used for clinical trials for cancer treatment (41, 42). CTSL protein levels were also reported to be downregulated by the treatment of cordycepin, a potential anti-cancer small molecule, in MDA-MB-231 (27). However, no too much progress has been made in TNBC (43). Identification of CTSL as a new potential therapeutic and prognostic target for TNBC is of great significance for TNBC patients.

In this study, we demonstrated that CTSL level is more highly expressed in TNBC tissues than that of paracancer tissues by western blotting and IHC, which was significantly associated with differentiated degree, TNM stage, tumor size, and lymphatic metastasis. We scored the IHC results of CTSL in paraffin breast cancer tissues to distinguish CTSL expression levels, where the score closest to the maximum point of the Yoden index was selected as the optimal threshold through receiver operating characteristic (ROC) curve analysis. Samples with scores below the critical value (score <6.3) were defined as low CTSL expressions, while samples with scores above the critical value (score >6.3) were defined as high CTSL expressions. We think that the optimal threshold value obtained by ROC is more objective, even though some studies considered an IHC score higher than 4 as a high expression (44). We further found that high CTSL expression predicted poor prognosis in TNBC patients, and CTSL expression in BC with Lymph node-positive revealed that a high expression of CTSL is correlated with a short DMFS and RFS. Moreover, overexpression of CTSL could significantly enhance the proliferation, migration, and invasion in the cells of MDA-MB-231 and MCF-7, while knockdown CTSL could significantly inhibit in MDA-MB-231 cells. These results imply that CTSL may be an effective biomarker for TNBC. Of course, it will be much better if have the *in vivo* data to support the conclusion as a biomarker. And we will do it in the future. Mechanistically, CTSL/CTSB was reported to regulate TGF-β production/signaling, which was required for the activation of fibroblasts and their promotion of invasive growth in human melanoma cells (45). Inflammatory BC (IBC) is the most aggressive and lethal form of BC, showing a poor prognosis and high infiltration of tumor-associated macrophages. El-Nadi et al. reported that CTSL and CD14+ monocytes-derived IL-10 play vital roles in the pathogenesis and their targeting might have therapeutic significances in IBC (46). Overall, CTSL which promoted the proliferation, migration, and invasion of MDA-MB-231 and MCF-7 may be associated with biological processes and signaling pathways, but it is still unclear for the potential mechanisms/pathways, which need to be further studied. Besides, CTSL is reported to contribute to tumor angiogenesis and its inhibition may have therapeutic significances in BC patients (47). The potential mechanisms/pathways BC angiogenesis need to be further studied.

In conclusion, CTSL is highly expressed in TNBC tissues and correlated with clinical progression. CTSL promotes the proliferation and migration/invasion in TNBC cells. High CTSL expression predicted poor prognosis in TNBC patients.

## Data availability statement

The original contributions presented in the study are included in the article/supplementary material. Further inquiries can be directed to the corresponding authors.

## Ethics statement

The work was approved by the Ethical Committee of Southwest Medical University and Huai’an People’s Hospital. The patients/participants provided their written informed consent to participate in this study.

## Author contributions

LZ, YZha, and YZhu collected the samples and did IHC. JY, CW, JC, TL, XL, and PZ did cell culture, western blotting, RT-PCR, assays of cell growth, migration and invasion, and data analysis. JF, SS, and LZ designed and supervised the project. LZ and JF wrote and edited the manuscript. All authors contributed to the article and approved the submitted version.
